# Cardiovascular Disease as a Predictor of Dementia: A Cross‐National Ecological Study of 204 Countries

**DOI:** 10.1002/hsr2.71179

**Published:** 2025-08-27

**Authors:** Wenpeng You

**Affiliations:** ^1^ Adelaide Medical School the University of Adelaide Adelaide South Australia Australia; ^2^ School of Nursing and Midwifery Western Sydney University Sydney New South Wales Australia

**Keywords:** cardiovascular disease, dementia, epidemiology, global health, health disparities, incidence correlation

## Abstract

**Background and Aims:**

Cardiovascular disease (CVD) and dementia represent two of the most pressing global health challenges, particularly in low‐ and middle‐income countries (LMICs). While vascular pathology is increasingly recognized as a contributor to cognitive decline, few studies have systematically explored the global association between CVD and dementia using standardized, population‐level data. This study aimed to investigate the relationship between CVD and dementia incidence across 204 countries, stratified by economic status, development level, and geographic region.

**Methods:**

Age‐standardized incidence rates for cardiovascular disease (CVD) and dementia in 2021 were sourced from the Global Burden of Disease Study. To examine global and regional associations, bivariate analyses (Pearson and Spearman correlations) and partial correlations were conducted, adjusting for ageing, economic affluence, genetic predisposition, and urbanization. Linear and multivariate stepwise regression models were applied to estimate the extent to which CVD incidence contributes to dementia incidence at the population level.

**Results:**

Globally, CVD incidence was significantly associated with dementia incidence (Pearson *r* = 0.777; Spearman *ρ* = 0.868; *p* < 0.001). CVD explained approximately 43.0% of the variance in dementia incidence at the population level (*r*² = 0.4303), even after adjusting for key confounders. The association was notably stronger in low‐ and middle‐income countries (LMICs) and developing regions. Among CVD subtypes, peripheral arterial disease (*β* = 0.903), cardiomyopathy (*β* = 0.869), and atrial fibrillation (*β* = 0.708) demonstrated the strongest independent associations with dementia incidence. Conversely, rheumatic heart disease exhibited a negative association.

**Conclusion:**

This study demonstrates a robust population‐level association between CVD and dementia across global settings, particularly in resource‐limited regions. Findings underscore the importance of integrated public health approaches targeting shared vascular risk factors. Given the ecological design, further individual‐level, longitudinal research is needed to clarify causal pathways and inform targeted interventions.

**Abbreviations**
ACD: Asia Cooperation Dialogue
AFRO: African Region (WHO)
AMRO: Region of the Americas (WHO)
APEC: Asia‐Pacific Economic Cooperation
ASIR: Age‐Standardized Incidence Rate
CVD: cardiovascular disease
EEA: European Economic Area
EMRO: Eastern Mediterranean Region (WHO)
EOL: English as Official Language
EU: European Union
GBD: Global Burden of Disease
GDP: Gross Domestic Product
IHME: Institute for Health Metrics and Evaluation
LAC: Latin America and the Caribbean
LA: Latin America
LMICs: Low‐ and Middle‐Income Countries
NCDs: Non‐Communicable Diseases
OECD: Organisation for Economic Co‐operation and Development
PPP: purchasing power parity
SADC: Southern African Development Community
SCO: Shanghai Cooperation Organisation
SDGs: Sustainable Development Goals
SEARO: South‐East Asia Region (WHO)
UN: United Nations
WHO: World Health Organization
WPRO: Western Pacific Region (WHO)

## 1 Background

Dementia is a leading cause of disability, dependency, and premature mortality among older adults worldwide, affecting an estimated 55 million individuals globally [1]. This number is projected to double by 2050 due to rapid population ageing [1]. Alzheimer's disease and other dementias exert profound personal, social, and economic impacts, particularly as health systems struggle to meet the needs of ageing populations [2]. Identifying modifiable risk factors for dementia remains a public health priority, as preventive strategies could delay onset or reduce disease burden [3].

Cardiovascular disease (CVD) has emerged as a key, potentially modifiable risk factor in the development of dementia [4, 5]. The vascular hypothesis of dementia is increasingly supported by evidence indicating that mechanisms underlying CVD, such as atherosclerosis, chronic inflammation, endothelial dysfunction, and cerebral hypoperfusion, also contribute to neurodegeneration and cognitive decline [4, 6]. These overlapping biological pathways, which frequently co‐occur in clinical settings, suggest a shared etiology that supports the need for integrated management strategies [7].

Alzheimer's disease is the most common form of dementia, accounting for approximately 60%–70% of cases, while vascular dementia is generally considered the second most prevalent, with estimates ranging from 15% to 30% of diagnoses [1]. However, these figures are derived largely from clinical classifications rather than robust epidemiological evidence [8]. As such, while they highlight the clinical recognition of vascular contributions, they may not accurately reflect the true population‐level impact of CVD on dementia incidence or its potential for prevention [9].

Additionally, mixed dementia, characterized by the co‐occurrence of vascular and Alzheimer's pathology, is reported in 25%–35% of cases, further highlighting the substantial and often underestimated role of CVD in the global dementia burden [10, 11].

Much of the existing literature on the relationship between CVD and dementia originates from longitudinal studies conducted in high‐income countries. However, there is an urgent need to investigate this association in a global context, particularly in low‐ and middle‐income countries (LMICs) [4, 12], where both conditions are becoming increasingly prevalent. Demographic transitions and shifting lifestyles in LMICs are driving a double burden of chronic disease [12], yet these regions often lack adequate healthcare infrastructure, diagnostic capacity, and preventive services [9]. Understanding how CVD and dementia co‐occur and interact in diverse socioeconomic and health system contexts is therefore essential for informing effective, equitable global health strategies.

Despite the growing burden of CVD and dementia worldwide, few studies have systematically examined their association using standardized, population‐level data across diverse global settings [3]. Existing research rarely accounts for regional variations in healthcare access, demographic composition, or stages of epidemiological transition, all of which may influence the strength and consistency of this relationship [13].

To address this gap, this study analyzed the association between CVD and dementia incidence across 204 countries, employing correlation and regression analyses stratified by economic and geopolitical groupings. This analysis offers new empirical insights into how consistently and strongly CVD correlates with dementia across different global contexts, helping to identify cardiovascular conditions and regions with the highest dementia risk.

The study findings support the need for integrated cardiovascular and cognitive health strategies, particularly in LMICs where health systems are under strain and non‐communicable disease burdens are rising.

## 2 Materials and Methods

### 2.1 Data Sources

This study utilized global epidemiological data from the Global Burden of Disease Study 2021 (GBD 2021), a comprehensive data set developed by the Institute for Health Metrics and Evaluation (IHME) at the University of Washington [14]. The GBD provides internationally comparable, age‐standardized estimates of disease burden by integrating data from administrative records, population‐based surveys, disease registries, and vital statistics. These diverse sources are synthesized using DisMod‐MR, an advanced Bayesian meta‐regression modeling tool that ensures consistency and comparability across countries and time periods [14].

For this analysis, age‐standardized incidence rates (ASIRs) for CVD ADOD, were extracted for both sexes combined. All estimates were reported per 100,000 population and reflect data from the year 2021. The IHME provided data on 16 specific cardiovascular variables, which included 15 site specific diagnose and 1 grouped diagnoses.

To investigate regional and socioeconomic differences in the association between CVD and dementia, countries were grouped into 23 predefined categories based on development level, health system context, and geopolitical affiliations. These groupings included the World Bank's economic classifications of low‐income, lower‐middle‐income, upper‐middle‐income, and high‐income economies [15]. Countries were also classified by developmental status using the United Nations' framework distinguishing developed and developing nations [16].

Additionally, the study applied the World Health Organization's regional framework, which categorizes countries into six major regions: the African Region (AFRO), Region of the Americas (AMRO), Eastern Mediterranean Region (EMRO), European Region (EURO), South‐East Asia Region (SEARO), and Western Pacific Region (WPRO) [17]. Further geopolitical and regional entities were included to enhance contextual analysis. These included the Arab World, Asia‐Pacific Economic Cooperation (APEC), European Union (EU), Organisation for Economic Co‐operation and Development (OECD), Shanghai Cooperation Organisation (SCO), Southern African Development Community (SADC), and the Economic Commission for Latin America and the Caribbean (ECLAC).

This classification framework allowed for comprehensive comparative analyses, offering insight into global patterns and enabling direct comparisons between LMICs and high‐income nations in the context of CVD and dementia incidence.

### 2.2 Study Design and Statistical Analysis

This study included 16 CVD variables based on classifications from the Global Burden of Disease Study 2021. These consisted of 15 site‐specific diagnoses and one composite measure representing total CVD. Each condition was treated as an independent predictor to examine its association with dementia incidence. The site‐specific conditions included rheumatic heart disease, ischemic heart disease, and stroke, which was further divided into ischemic stroke, intracerebral hemorrhage, and subarachnoid hemorrhage. Additional conditions encompassed non‐rheumatic valvular heart diseases such as calcific aortic and degenerative mitral valve diseases, cardiomyopathy and myocarditis including myocarditis, pulmonary arterial hypertension, and atrial fibrillation and flutter, as well as lower extremity peripheral arterial disease and endocarditis.

In addition to analyzing individual conditions, the study also examined broader CVD groupings including cardiomyopathy and myocarditis, non‐rheumatic valvular diseases, and total CVD. These clinically and pathophysiologically related clusters allowed for a more comprehensive evaluation of the cardiovascular contribution to dementia incidence across different countries.

To assess the strength and direction of the association between CVD and dementia incidence, bivariate analyses were first conducted using Pearson's correlation coefficient (*r*) and Spearman's rank correlation coefficient (*ρ*), which measure linear and monotonic relationships, respectively. To further examine the relationship, simple and multiple linear regression models were applied, with dementia incidence as the dependent variable and CVD incidence as the primary independent variable. The multiple regression models adjusted for key confounders, including ageing (measured by life expectancy at birth), economic affluence (GDP per capita in purchasing power parity), genetic predisposition (Biological State Index), and urbanization (percentage of the population living in urban areas).

To assess whether the strength of association between CVD and dementia differed across global contexts, Fisher's *r*‐to‐*z* transformation was applied to compare correlation coefficients across predefined country groupings, including high‐income versus LIMCs and developed versus developing nations. Additional subgroup analyses were conducted based on income level, development status, and World Health Organization (WHO) regions to further explore regional and socioeconomic variation.

All statistical analyses were conducted using IBM SPSS Statistics (Version 30.0; IBM Corp., Armonk, NY, USA). The analytical methods included Pearson and Spearman correlations, partial correlations (adjusting for ageing, economic affluence, genetic predisposition, and urbanization), and both simple and multivariate stepwise linear regression. Fisher's *r*‐to‐*z* transformation was used for group comparisons. All tests were two‐sided, with statistical significance set at *p* < 0.05, and results with *p* < 0.01 highlighted where applicable. The study design and statistical methods were guided by standard references and aligned with the SAMPL (Statistical Analyses and Methods in the Published Literature) guidelines. Reporting practices were further informed by the recommendations of Assel et al. [18], with an emphasis on effect sizes, confidence intervals, and appropriate interpretation of findings.

**Figure 1:**
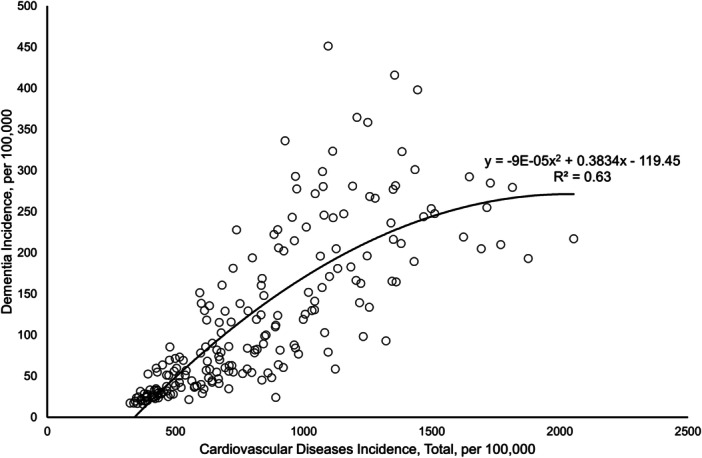
Curvilinear relationship between a range of cardiovascular diseases and dementia incidence.

**Figure 2:**
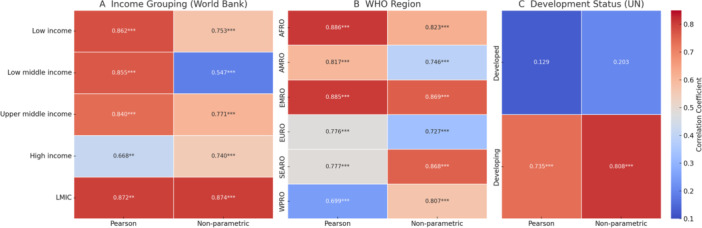
Stratified heat maps of cardiovascular–dementia correlations by World Bank Income Groups, WHO Regions, and UN development status. (A) Income Grouping (World Bank) (B) WHO Region (C) Development Status (UN).

Visual analyses were conducted to support key findings. A scatter plot with quadratic regression (Figure 1) illustrates the global CVD–dementia relationship. Stratified heat maps (Figure 2) display correlation strength across World Bank income groups, WHO regions, and UN development status, complementing subgroup results in Table 3.

## 3 Results

Figure 1 presents a scatter plot illustrating the nonlinear relationship between total CVD incidence and dementia incidence, both expressed per 100,000 population. A quadratic regression model was fitted to the data, yielding the equation *y* = −9E − 05x^2^ + 0.3834x − 119.45, with an *R* ^2^ value of 0.63. This indicates that 63% of the *between‐country variance* in dementia incidence can be explained by the incidence of CVD.

The curvilinear pattern suggests a positive association between CVD and dementia incidence at lower to moderate levels of total CVD burden, where increases in CVD are associated with sharp increases in dementia incidence. However, the negative coefficient of the squared term implies a decelerating trend at higher levels of CVD incidence, indicating a potential plateau in dementia incidence despite further increases in CVD burden. This pattern may reflect underlying saturation effects or competing risks that attenuate the strength of the association in populations with very high CVD incidence.

These findings highlight the importance of cardiovascular health as a significant predictor of dementia incidence and suggest that public health strategies targeting cardiovascular disease prevention may have broader implications for reducing dementia burden globally.

A comprehensive correlation matrix was constructed to examine associations between dementia and a range of cardiovascular, neurological, demographic, and socioeconomic variables (File S1: Combined Correlation Matrix of Cardiovascular, Neurological, Demographic, and Socioeconomic Variables).

The results revealed that dementia was most strongly associated with atrial fibrillation and flutter (*r* = 0.945, *p* < 0.001), lower extremity peripheral arterial disease (*r* = 0.959, *p* < 0.001), cardiomyopathy and myocarditis (*r* = 0.927, *p* < 0.001), and myocarditis alone (*r* = 0.916, *p* < 0.001). These findings suggest that underlying cardiovascular dysfunction, particularly involving atrial and peripheral arterial conditions, may play a critical role in dementia pathogenesis.

Demographic and genetic factors also exhibited robust correlations with dementia. Ageing (*r* = 0.832, *p* < 0.001) and genetic predisposition (*r* = 0.853, *p* < 0.001) were among the strongest non‐cardiovascular correlates. Economic affluence (*r* = 0.779, *p* < 0.001) also demonstrated a notable positive relationship with dementia, potentially reflecting disparities in health behaviors, access to healthcare, or diagnostic sensitivity in high‐income populations.

Other cardiovascular and cerebrovascular conditions, including ischemic heart disease (*r* = 0.597, *p* < 0.001), ischemic stroke (*r* = 0.685, *p* < 0.001), and stroke overall (*r* = 0.598, *p* < 0.001), showed moderate‐to‐strong positive associations with dementia. In contrast, rheumatic heart disease was negatively correlated with dementia (*r* = –0.743, *p* < 0.001), possibly due to its higher prevalence in low‐income settings where dementia detection and reporting may be limited.

Urban advantage was modestly correlated with dementia (*r* = 0.530, *p* < 0.001), suggesting that urban living may be linked to increased risk, although the directionality and causality of this relationship require further exploration.

Collectively, these findings reinforce the multifactorial etiology of dementia, emphasizing the significant contributions of cardiovascular health, aging, genetics, and socioeconomic context. The results support integrative strategies for dementia prevention that address vascular risk reduction alongside demographic and social determinants of health.

Table 1 outlines the strength of associations between dementia and a range of cardiovascular and cerebrovascular conditions, using Pearson, Spearman, and partial correlation coefficients. The partial correlations adjust for key confounding variables: ageing, economic affluence, genetic predisposition, and urban advantage.

**Table 1:** Associations between cardiovascular/cerebrovascular conditions and Alzheimer's disease.

Disease correlation with dementia	Pearson	Spearman	Partial correlation	*n*
Atrial fibrillation and flutter	0.876**	0.945**	0.739**	204
Cardiomyopathy and myocarditis	0.927**	0.916**	0.851**	204
Cardiovascular diseases	0.777**	0.868**	0.656**	204
Endocarditis	0.781**	0.802**	0.560**	204
Intracerebral hemorrhage	0.028	0.066	0.182*	204
Ischemic heart disease	0.422**	0.597**	0.236**	204
Ischemic stroke	0.685**	0.823**	0.665**	204
Lower extremity peripheral arterial disease	0.928**	0.959**	0.840**	204
Myocarditis	0.927**	0.916**	0.851**	204
Non‐rheumatic calcific aortic valve disease	0.833**	0.924**	0.710**	204
Non‐rheumatic degenerative mitral valve disease	0.674**	0.863**	0.541**	204
Non‐rheumatic valvular heart disease	0.825**	0.928**	0.707**	204
Pulmonary Arterial Hypertension	0.371**	0.355**	0.432**	204
Rheumatic heart disease	−0.700**	‐0.743**	‐0.264**	204
Stroke	0.598**	0.709**	0.615**	204
Subarachnoid hemorrhage	0.492**	0.620**	0.289**	204
Ageing	0.743**	0.832**	Control variable	175
Economic Affluence	0.605**	0.779**	Control variable	175
Genetic Predisposition	0.609**	0.853**	Control variable	175
Urban Advantage	0.505**	0.530**	Control variable	175

*Note:* Aging in females was measured by life expectancy at birth (World Bank, 2018). Economic affluence was represented by GDP per capita in purchasing power parity (PPP), also from the World Bank (2018). Genetic predisposition was indexed using the Biological State Index (Ibs), reflecting the accumulation of dementia‐related genetic risk due to relaxed natural selection (You & Henneberg, 2022). Urban living was measured by the percentage of the population residing in urban areas (World Bank, 2018).

* Correlation is significant at the 0.05 level (2‐tailed).

** Correlation is significant at the 0.01 level (2‐tailed).

The analysis reveals that cardiomyopathy and myocarditis (*r* = 0.851) and myocarditis (*r* = 0.851) show the strongest independent associations with dementia, followed closely by lower extremity peripheral arterial disease (*r* = 0.840) and atrial fibrillation and flutter (*r* = 0.739). These findings suggest that certain cardiovascular conditions, especially those involving inflammation and impaired circulation, may influence dementia risk independently of demographic factors.

Other notable associations include non‐rheumatic calcific aortic valve disease (*r* = 0.710), non‐rheumatic valvular heart disease (*r* = 0.707), and ischemic stroke (*r* = 0.665), which continue to exhibit strong correlations after adjustment, supporting a vascular hypothesis of dementia.

More moderate yet statistically significant correlations were observed for cardiovascular diseases overall (*r* = 0.656), stroke (*r* = 0.615), endocarditis (*r* = 0.560), and non‐rheumatic degenerative mitral valve disease (*r* = 0.541). These conditions likely contribute to cumulative cerebrovascular burden, further increasing dementia risk.

Weaker associations were noted for ischemic heart disease (*r* = 0.236), subarachnoid hemorrhage (*r* = 0.289), and pulmonary arterial hypertension (*r* = 0.432), which may influence dementia through indirect pathways.

Conversely, rheumatic heart disease exhibited a significant negative partial correlation with dementia (*r* = –0.264). This inverse relationship could reflect sociocultural and healthcare access factors that differentially influence dementia diagnosis or disease reporting in affected populations.

Overall, the findings suggest a robust and multifaceted connection between cardiovascular health and dementia, emphasizing the importance of managing vascular risk factors in dementia prevention strategies.

Multivariate stepwise regression analyses were conducted to identify the strongest cardiovascular and demographic predictors of dementia. As presented in Table 2, lower extremity peripheral arterial disease emerged as the most robust independent predictor, with a standardized coefficient of *β* = 0.903, explaining a large proportion of variance (adjusted *R*² = 0.877), suggesting a strong population‐level association. Similarly, cardiomyopathy and myocarditis showed a strong and significant association with dementia incidence (*β* = 0.869, *p* < 0.001), yielding an adjusted *R*² of 0.868. When myocarditis was examined independently, it produced identical results (*β* = 0.869, *p* < 0.001; adjusted *R*² = 0.868), suggesting it may drive much of the predictive strength within the broader category.

**Table 2:** Multivariate stepwise regression results predicting Alzheimer's disease and other dementia incidence: standardized coefficients, significance, and adjusted *R*² across cardiovascular predictors.

Enter method				Stepwise method			
Model	Variable	Standardized (*β*)	Significance (*p*)	Model	Variable	Adjusted *R* ^2^	Significance (*p*)
1	Ageing	0.377	< 0.01	1	Atrial fibrillation and flutter	0.757	< 0.01
	Economic affluence	−0.082	0.154	2	Ageing	0.785	< 0.01
	Genetic predisposition	−0.152	0.042		Economic affluence	Insignificant	
	Urban advantage	0.090	0.051		Urban advantage	Insignificant	
	Atrial fibrillation and flutter	0.708	< 0.01		Genetic predisposition	Insignificant	
1	Ageing	0.348	< 0.01	1	Cardiomyopathy and myocarditis	0.859	< 0.01
	Economic affluence	−0.139	0.010	2	Urban advantage	0.868	< 0.01
	Genetic predisposition	−0.249	< 0.01		Ageing	Insignificant	
	Urban advantage	0.125	< 0.01		Economic affluence	Insignificant	
	Cardiomyopathy and myocarditis	0.869	< 0.01		Genetic predisposition	Insignificant	
1	Ageing	0.634	< 0.01	1	Cardiovascular diseases	0.605	< 0.01
	Economic affluence	0.079	0.201	2	Ageing	0.714	< 0.01
	Genetic predisposition	−0.362	< 0.01	3	Genetic predisposition	0.746	< 0.01
	Urban advantage	0.021	0.68		Economic affluence	Insignificant	
	Cardiovascular diseases	0.577	< 0.01		Urban advantage	Insignificant	
1	Ageing	0.517	< 0.01	1	Endocarditis	0.595	< 0.01
	Economic affluence	−0.063	0.37	2	Ageing	0.686	< 0.01
	Genetic predisposition	−0.128	0.161		Economic affluence	Insignificant	
	Urban advantage	0.072	0.201		Urban advantage	Insignificant	
	Endocarditis	0.511	< 0.01		Genetic predisposition	Insignificant	
1	Ageing	0.593	< 0.01	1	Ageing	0.542	< 0.001
	Economic affluence	−0.288	< 0.01		Economic affluence	Insignificant	
	Genetic predisposition	−0.078	0.338		Urban advantage	Insignificant	
	Urban advantage	0.047	0.549		Genetic predisposition	Insignificant	
	Intracerebral hemorrhage	0.158	< 0.01		Intracerebral hemorrhage	Insignificant	
1	Ageing	0.530	< 0.01	1	Ageing	0.542	< 0.01
	Economic affluence	−0.191	< 0.01	2	Ischemic heart disease	0.557	< 0.01
	Genetic predisposition	−0.163	< 0.05	3	Genetic predisposition	0.571	< 0.01
	Urban advantage	0.077	0.242		Economic affluence	Insignificant	
	Ischemic heart disease	0.197	< 0.01		Urban advantage	Insignificant	
1	Ageing	0.504	< 0.01	1	Ageing	0.542	< 0.01
	Economic affluence	‐0.061	0.383	2	Ischemic stroke	0.722	< 0.01
	Genetic predisposition	−0.127	0.126	3	Genetic predisposition	0.752	< 0.01
	Urban advantage	0.109	0.085		Economic affluence	Insignificant	
	Ischemic stroke	0.511	< 0.01		Urban advantage	Insignificant	
1	Ageing	0.381	< 0.01	1	Lower extremity peripheral arterial disease	0.868	< 0.01
	Economic affluence	−0.121	< 0.05	2	Ageing	0.87	< 0.05
	Genetic predisposition	−0.265	< 0.01	3	Economic affluence	0.877	< 0.01
	Urban advantage	0.096	< 0.05		Genetic predisposition	Insignificant	
	Peripheral arterial disease	0.903	< 0.01		Urban advantage	Insignificant	
1	Ageing	0.348	< 0.01	1	Myocarditis	0.859	< 0.01
	Economic affluence	−0.139	< 0.01	2	Urban advantage	0.868	< 0.01
	Genetic predisposition	−0.249	< 0.01		Ageing	Insignificant	
	Urban advantage	0.125	< 0.01		Economic affluence	Insignificant	
	Myocarditis	0.869	< 0.01		Genetic predisposition	Insignificant	
1	Ageing	0.412	< 0.01	1	Non‐rheumatic calcific aortic valve disease	0.716	< 0.01
	Economic affluence	−0.186	< 0.01	2	Ageing	0.783	< 0.01
	Genetic predisposition	−0.011	0.885	3	Economic affluence	0.791	< 0.01
	Urban advantage	0.068	0.14		Genetic predisposition	Insignificant	
	Calcific aortic valve disease	0.683	< 0.01		Urban advantage	Insignificant	
1	Ageing	0.637	< 0.01	1	Ageing	0.542	< 0.01
	Economic affluence	−0.064	0.346	2	Non‐rheumatic degenerative mitral valve disease	0.702	< 0.01
	Genetic predisposition	−0.149	0.095		Economic affluence	Insignificant	
	Urban advantage	0.075	0.168		Genetic predisposition	Insignificant	
	Degenerative mitral valve disease	0.469	< 0.01		Urban advantage	Insignificant	
1	Ageing	0.459	< 0.01	1	Non‐rheumatic valvular heart disease	0.724	< 0.01
	Economic affluence	−0.173	< 0.01	2	Ageing	0.795	< 0.01
	Genetic predisposition	−0.068	0.349	3	Economic affluence	0.801	< 0.05
	Urban advantage	0.082	0.066		Genetic predisposition	Insignificant	
	Valvular heart disease	0.675	< 0.01		Urban advantage	Insignificant	
1	Ageing	0.732	< 0.01	1	Ageing	0.542	< 0.01
	Economic affluence	0.059	0.435	2	Pulmonary arterial hypertension	0.615	< 0.01
	Genetic predisposition	−0.129	0.199		Economic affluence	Insignificant	
	Urban advantage	0.073	0.240		Genetic predisposition	Insignificant	
	Pulmonary arterial hypertension	0.281	< 0.01		Urban advantage	Insignificant	
1	Ageing	0.689	< 0.01	1	Ageing	0.542	< 0.01
	Economic affluence	0.001	0.987	2	Rheumatic heart disease	0.567	< 0.01
	Genetic predisposition	−0.26	0.019	3	Genetic predisposition	0.58	< 0.05
	Urban advantage	0.012	0.854		Economic affluence	Insignificant	
	Rheumatic heart disease	−0.331	< 0.01		Urban advantage	Insignificant	
1	Ageing	0.81	< 0.01	1	Ageing	0.542	< 0.01
	Economic affluence	0.116	0.067	2	Stroke	0.688	< 0.01
	Genetic predisposition	−0.421	< 0.01	3	Genetic predisposition	0.73	< 0.01
	Urban advantage	0.062	0.23	4	Economic affluence	0.737	< 0.05
	Stroke	0.492	< 0.01		Urban advantage	Insignificant	
1	Ageing	0.78	< 0.01	1	Ageing	0.542	< 0.01
	Economic affluence	0.097	0.217	2	Subarachnoid hemorrhage	0.571	< 0.01
	Genetic predisposition	−0.321	< 0.01	3	Genetic predisposition	0.592	< 0.01
	Urban advantage	0.049	0.441		Economic affluence	Insignificant	
	Subarachnoid hemorrhage	0.282	< 0.01		Urban advantage	Insignificant	

*Note:* Aging in females was measured by life expectancy at birth (World Bank, 2018); Economic affluence was represented by GDP per capita in purchasing power parity (PPP), also from the World Bank (2018); Genetic predisposition was indexed using the Biological State Index (Ibs), reflecting the accumulation of dementia‐related genetic risk due to relaxed natural selection (You & Henneberg, 2022); Urban living was measured by the percentage of the population residing in urban areas (World Bank, 2018).

*Correlation is significant at the 0.05 level (2‐tailed); **Correlation is significant at the 0.01 level (2‐tailed).

Several other cardiovascular conditions also demonstrated statistically significant positive associations with dementia incidence. These included non‐rheumatic valvular heart disease (*β* = 0.675, *p* < 0.001; adjusted *R*² = 0.801), non‐rheumatic calcific aortic valve disease (*β* = 0.683, *p* < 0.001; adjusted *R*² = 0.791), and atrial fibrillation and flutter (*β* = 0.708, *p* < 0.001; adjusted *R*² = 0.785). Ischemic stroke (*β* = 0.511, *p* < 0.001; adjusted *R*² = 0.752), overall cardiovascular disease (*β* = 0.577, *p* < 0.001; adjusted *R*² = 0.746), and stroke as a broader category (*β* = 0.492, *p* < 0.001; adjusted *R*² = 0.737) also emerged as meaningful predictors.

In contrast, rheumatic heart disease demonstrated a significant negative association with dementia incidence (*β* = –0.331, *p* < 0.001; adjusted *R*² = 0.580). This inverse relationship may reflect differences in population demographics, comorbidity profiles, or healthcare infrastructure across countries where rheumatic heart disease remains more prevalent.

Across all models, ageing consistently remained a significant and positive predictor of dementia incidence. However, once cardiovascular variables were included in the models, other demographic factors such as economic affluence, genetic predisposition, and urban advantage were frequently rendered statistically insignificant. This suggests that specific cardiovascular disease burdens may have a more direct and substantial influence on dementia outcomes than broader contextual or socioeconomic indicators.

These findings highlight a strong association between cardiovascular and cerebrovascular conditions and dementia incidence at the population level. They underscore the potential benefit of integrated public health approaches that address vascular health in relation to cognitive outcomes.

Table 3 presents the Pearson and Spearman correlation coefficients examining the association between CVD and dementia incidence across various country groupings. Stratified analyses by World Bank income classifications revealed strong correlations in all groups, with the highest Pearson correlation in low‐income countries (*r* = 0.862, *ρ* = 0.753, *p* < 0.01) and slightly lower correlations in high‐income countries (*r* = 0.668, *ρ* = 0.740, *p* < 0.01). A Fisher's *Z*‐test confirmed that the correlation was significantly stronger in LMICs compared to high‐income countries (Pearson's *z* = –3.56, *p* < 0.01; Spearman's *z* = –2.66, *p* < 0.01), indicating that the association may be more pronounced in resource‐constrained settings.

**Table 3:** Bivariate correlation between cardiovascular disease and dementia incidence across global groupings.

	Dementia incidence		
Country groupings	Pearson	Non‐parametric	*n*
Worldwide	0.656***	0.868***	204
World Bank income classifications			
Low income	0.862***	0.753***	50
Low‐middle‐income	0.855***	0.547***	49
Upper middle income	0.840***	0.771***	55
High income	0.668**	0.740***	70
LMIC	0.872**	0.874***	134
Fisher A‐to‐Z: high income vs LMICs in Pearson's *r* (*z* = −3.56, *p* < 0.001) and in non‐parametric (*z* = −2.66, *p* < 0.010)			
UN common practice			
Developed	0.129	0.203	49
Developing	0.735***	0.808***	155
Fisher A‐to‐Z: developed vs developing in Pearson's *r* (*z* = −4.81, *p* < 0.001) and in non‐parametric (*z* = −5.44, *p* < 0.001)			
WHO Regions			
AFRO	0.886***	0.823***	47
AMRO	0.817***	0.746***	38
EMRO	0.885***	0.869***	22
EURO	0.776***	0.727***	50
SEARO	0.777***	0.868***	11
WPRO	0.699***	0.807***	32
Countries grouped based on various factors			
ACD	0.452*	0.404***	30
APEC	0.729***	0.644***	19
Arab World	0.643**	0.842***	20
EEA	0.379*	0.395*	29
EOL	0.816***	0.906**	57
EU	0.356	0.308	27
LA	0.831***	0.699***	24
LAC	0.767***	0.683**	35
OECD	0.568***	0.684***	37
SADC	0.958***	0.868***	16
SCO	0.795***	0.652***	26

*Note:* Cardiovascular disease incidence rates and dementia incidence rates (including Alzheimer's disease and other dementias), for both sexes, were obtained from the Institute for Health Metrics and Evaluation (IHME), University of Washington. Incidence rates are reported as the number of new cases per 100,000 population in 2021.

* *p *< 0.05

** *p* < 0.01

*** *p* < 0.001.

Similarly, based on the UN development classification, developing countries exhibited a substantially stronger association (*r* = 0.735, *ρ* = 0.808, *p* < 0.01) than developed countries, which showed a nonsignificant and weak relationship (*r* = 0.129, *ρ* = 0.203, *p* > 0.05). The difference was statistically significant (Pearson's *z* = –4.81, *p* < 0.01; Spearman's *z* = –5.44, *p* < 0.01), further highlighting regional disparities.

Analyses across WHO regions revealed consistently strong and significant associations in all six regions, with the AFRO and EMRO showing particularly high correlations (*r* = 0.886 and 0.885, respectively).

When grouped by geopolitical and economic alliances (e.g., OECD, SCO, EU, Arab World), most regions demonstrated moderate to strong correlations. Notably, the SADC exhibited the highest correlation across all groups (*r* = 0.958, *ρ* = 0.868, *p *< 0.01), while EU presented weaker and nonsignificant associations (*r* = 0.356, *ρ* = 0.308, *p* > 0.05).

Overall, the findings underscore a robust and consistent global association between cardiovascular disease and dementia incidence, with stronger correlations in lower‐income and developing regions. This may reflect disparities in healthcare infrastructure, preventive care access, and comorbid risk factor management, warranting targeted public health interventions.

Figure 2 presents stratified heat maps that visually illustrate the variation in correlation strength across World Bank income groups, WHO regions, and undefined development status, reinforcing the subgroup patterns reported in Table 3.

## 4 Discussion

This study provides compelling global evidence of a positive and statistically significant association between CVD incidence and dementia incidence, based on age‐standardized data from 204 countries and territories. Both parametric (Pearson's r) and non‐parametric (Spearman) correlation analyses consistently demonstrated that higher rates of CVD are associated with increased dementia incidence at the population level. These findings support the vascular hypothesis of dementia, which suggests that vascular dysfunction plays a role in cognitive decline, including Alzheimer's disease [4, 6].

In 2021, approximately 57 million people were living with dementia worldwide, with over 60% residing in LMICs [19]. Each year, nearly 10 million new cases are diagnosed, disproportionately affecting resource‐constrained regions. Notably, this study found that the strength of the association between CVD and dementia varied significantly across global regions and economic classifications. LMICs and developing nations exhibited the strongest correlations, as confirmed by significant Fisher's *z*‐tests comparing these groups with high‐income and developed countries [20]. This pattern suggests that in many lower‐resource settings, where vascular risk factors are often under‐managed, the observed associations may reflect a combination of high underlying disease burden and limited access to preventive care, though interpretations must go beyond statistical significance and consider contextual factors [4, 21]. Moreover, underfunded health systems may struggle with early detection, coordinated chronic disease management, and equitable access to preventive care, all of which may further intensify this dual burden amid ongoing epidemiological transitions [22].

The AFRO, EMRO, and SADC regions showed the strongest correlations. In these areas, both CVD and dementia are increasing rapidly [1, 23]. These findings underscore the urgent need for integrated public health strategies in these regions that simultaneously address cardiovascular and cognitive health by targeting shared risk factors and strengthening surveillance capacity [24].

Given the large sample size of 204 countries, many associations in this study achieved statistical significance. However, statistical significance alone does not imply clinical or practical relevance. For instance, although ischemic heart disease and subarachnoid hemorrhage were significantly associated with dementia, their effect sizes were modest and the proportion of variance explained was limited. These findings should be interpreted cautiously, with greater emphasis placed on the strength and consistency of associations rather than on p‐values alone when considering their implications for public health or clinical practice.

Conversely, weaker and sometimes nonsignificant associations were observed in several high‐income and developed settings, including EU. This attenuation may reflect more widespread implementation of cardiovascular prevention programs, greater healthcare access, early screening, and improved awareness of dementia‐related symptoms [4]. In these contexts, dementia incidence may increasingly be shaped by other pathways, such as age‐related neurodegeneration or genetic susceptibility, which may dilute the apparent influence of vascular conditions at the population level [25].

Partial correlation and regression analyses provided further insights. After adjusting for ageing, economic affluence, genetic predisposition, and urban living, several CVD conditions such as cardiomyopathy, myocarditis, atrial fibrillation, and peripheral arterial disease remained strongly associated with dementia incidence [6, 26, 27, 28]. This reinforces the hypothesis that distinct cardiovascular pathologies involving myocardial inflammation and impaired cerebral perfusion may play a particularly salient role in the development of dementia [29].

The curvilinear association, in which dementia incidence plateaus at higher levels of CVD, may reflect several underlying phenomena. One possibility is competing mortality risks, where individuals with severe CVD may die before reaching the age at which dementia typically manifests, thus reducing observed dementia incidence. Another explanation is diagnostic saturation, particularly in high‐CVD settings where healthcare systems may prioritize cardiovascular management over cognitive assessment, leading to underdiagnosis of dementia. Survivor bias may also play a role, whereby only healthier individuals with CVD live long enough to develop and be diagnosed with dementia. These factors likely interact, attenuating the observed association at the upper end of the CVD burden spectrum.

Beyond the epidemiological associations, mounting neuropathological and neuroimaging evidence supports the biological plausibility of cardiovascular contributions to dementia. Key vascular mechanisms such as atherosclerosis, chronic inflammation, and cerebral hypoperfusion can lead to structural and functional brain changes, including white matter hyperintensities, microinfarcts, cortical thinning, and disruptions in neurovascular coupling [30, 31]. These neuropathological alterations are increasingly recognized as core substrates of both vascular dementia and mixed dementia, where vascular pathology coexists with Alzheimer's‐type changes. The heterogeneity of dementia subtypes, especially in older adults, suggests that CVD may exert differential effects on cognitive decline depending on the type, severity, and chronicity of vascular insult. By situating our findings within this neurobiological framework, we underscore the importance of integrated prevention strategies that account not only for epidemiological risk patterns but also for the underlying brain mechanisms through which cardiovascular health influences cognitive aging.

From a public health perspective, these findings highlight the importance of upstream, integrated approaches to non‐communicable disease prevention [4]. Controlling modifiable vascular risk factors such as smoking, poor diet, physical inactivity, and hypertension may help reduce the incidence of both cardiovascular disease and dementia [2]. This is particularly crucial in LMICs, where capacity to manage comorbid conditions remains limited and where the population is ageing rapidly [32].

In contrast, rheumatic heart disease demonstrated a significant negative association with dementia incidence (*β* = –0.331, *p* < 0.001; adjusted *R*² = 0.580). This inverse relationship may reflect differences in population demographics, comorbidity profiles, or healthcare infrastructure across countries where rheumatic heart disease remains more prevalent. Underdiagnosis of dementia in these settings may contribute to this inverse association. Alternatively, infectious causes of CVD may involve different mechanisms than those linked to neurodegeneration.

The widely cited estimate that vascular dementia (VaD) accounts for 15%–30% of all dementia diagnoses reflects its clinical proportion among diagnosed cases rather than indicating an independent epidemiological association with dementia risk at the population level [8]. Mixed dementia, characterized by the co‐occurrence of vascular and Alzheimer's pathology, is reported in 25% to 35% of cases, further highlighting the substantial and often underestimated role of CVD in the global dementia burden [10, 11]. In this study, partial correlation analysis revealed that CVD was significantly and moderately strongly associated with the incidence of dementia, including Alzheimer's disease and other dementias (*r* = 0.656, *p* < 0.01). This correlation accounted for 43.03% of the country‐level variance in dementia incidence (*r*² = 0.4303), suggesting that nearly half of the variation in dementia burden may be statistically explained by CVD at the population level.

To further explore this relationship, multivariate stepwise regression was conducted using five key predictors: ageing, economic affluence, genetic predisposition, urbanization, and total CVD incidence. These variables collectively explained 74.6% of the observed cross‐national variance in dementia incidence (adjusted *R*² = 0.746), reinforcing the significance of cardiovascular and contextual demographic factors. These findings are consistent with the vascular hypothesis of dementia and suggest that CVD may be an important, though often underrecognized, factor contributing to dementia risk at the population level [4, 6].

## 5 Strengths and Limitations

While this study benefits from standardized data from the Global Burden of Disease Study 2021, covering over 200 countries, several limitations must be acknowledged.

First, the ecological and cross‐sectional design means findings reflect population‐level associations and cannot be applied to individuals. This introduces the risk of ecological fallacy, as within‐country variation and individual‐level confounders were not captured.

Second, causal inference is not possible, as temporal relationships between CVD and dementia cannot be established.

Third, although key covariates such as ageing, economic affluence, genetic predisposition, and urbanization were included, other relevant factors—such as healthcare access, education, and cultural attitudes toward dementia—were not accounted for, potentially contributing to residual confounding and regional disparities. Moreover, the proxy measures used may not fully represent these complex constructs. For instance, life expectancy does not reflect age distribution, GDP per capita may overlook inequality or access to care, and the Biological State Index (*I*_bs_), though standardized, is not yet widely validated in dementia research and should be interpreted with caution.

Fourth, while multiple CVD subtypes were analyzed, dementia was treated as a single aggregated outcome due to data constraints, limiting the ability to explore subtype‐specific pathways (e.g., Alzheimer's disease vs. vascular dementia). Future studies using disaggregated dementia data are needed to clarify these distinctions.

Finally, the study relies on modeled estimates from the IHME GBD, which may be subject to uncertainty and reporting bias, especially in LMICs where diagnostic infrastructure is limited. Variations in health system capacity, data quality, and diagnostic criteria may affect the accuracy of CVD and dementia incidence estimates. Despite these limitations, the study reveals consistent global patterns supporting a potential link between vascular health and dementia risk. Further research using individual‐level, longitudinal data is needed to confirm these associations and inform global dementia prevention efforts.

## 6 Public Health Implications

The findings of this study have important public health implications, particularly for LMICs and regions undergoing rapid epidemiological transitions. The strong association between CVD and dementia incidence across global contexts highlights the need for integrated prevention strategies targeting shared modifiable risk factors such as hypertension, diabetes, obesity, smoking, and physical inactivity. In LMICs, where healthcare systems often face resource constraints, strengthening cardiovascular prevention may yield dual benefits, reducing both CVD and dementia burden. These findings suggest that dementia prevention should be embedded within broader non‐communicable disease (NCD) control efforts.

The observed disparities in correlation strength between developing and developed nations highlight the urgency of improving vascular risk management in under‐resourced settings. This includes expanding access to routine screening, essential medications, and community‐based health promotion. Globally, the findings support intersectoral collaboration across cardiology, neurology, geriatrics, and public health. Policies to reduce the dementia burden must align with Sustainable Development Goals (SDGs), particularly Goal 3 on good health and well‐being. Investment in longitudinal surveillance, culturally tailored interventions, and equitable care access will be essential to translating these findings into effective and context‐specific public health action.

## 7 Conclusion

Reducing the future burden of dementia requires a shift toward holistic, cross‐sectoral strategies that prioritize cardiovascular health as a central component of lifelong cognitive well‐being. This approach is especially critical in LMICs, where both conditions are increasing and healthcare resources are often limited. The findings emphasize the interconnectedness of vascular and cognitive health and support the need for integrated prevention strategies targeting shared modifiable risk factors.

By drawing on standardized global data and diverse country groupings, the study highlights significant geographic and socioeconomic disparities in the burden of CVD and dementia. These disparities reinforce the importance of aligning cardiovascular and dementia prevention efforts, particularly in resource‐constrained settings where integrated approaches may offer substantial public health benefits. Given the strong population‐level association between cardiovascular health and cognitive outcomes, dementia prevention strategies may be enhanced by targeting vascular risk factors.

Nevertheless, due to the study's ecological design and reliance on modeled, population‐level data, findings should be interpreted with caution. Future longitudinal studies using individual‐level data and more granular classification of dementia subtypes are essential to validate and deepen understanding of these global associations.

## Author Contributions

**Wenpeng You:** conceptualization, investigation, funding acquisition, writing – original draft, writing – review and editing, visualization, validation, methodology, software, formal analysis, project administration, data curation, resources. The author has read and approved the final version of the manuscript. The corresponding author, Dr. Wenpeng You, had full access to all of the data in this study and takes complete responsibility for the integrity of the data and the accuracy of the data analysis.

## Ethics Statement

This study did not involve research with individual human participants or animals. All data used were publicly available and obtained from Institute for Health Metrics and Evaluation and official websites of United Nations (UN) agencies. According to the *National Statement on Ethical Conduct in Human Research* (2007, updated 2018), the University of Adelaide classifies research as exempt from ethical review when it involves only negligible risk and uses existing, non‐identifiable data about human beings (Reference No. XXXX, Details to be provided once this manuscript is accepted.).

## Consent

The author has nothing to report.

## Conflicts of Interest

The author declares no conflicts of interest.

## GEN AI Use Statement

During initial preparation of this manuscript, the author used ChatGPT 4.0 to enhance readability and language, without replacing key authoring tasks. After utilising this tool, the author edited the text, taking full responsibility for the publication's content.

## Transparency Statement

The sole author Wenpeng You affirms that this manuscript is an honest, accurate, and transparent account of the study being reported; that no important aspects of the study have been omitted; and that any discrepancies from the study as planned (and, if relevant, registered) have been explained.

## Supporting information


**SF 1 Table:** Combined Correlation Matrix of Cardiovascular, Neurological, Demographic, and Socioeconomic Variables (Pearson correlations shown above the diagonal; Spearman correlations shown below the diagonal).

## Data Availability

The data supporting the findings of this study are publicly available and have been described in detail in the *Materials and Methods* section. All data were obtained from official websites of international organizations, including the Institute for Health Metrics and Evaluation (IHME), the World Bank, and the United Nations. These data are freely accessible and were used in accordance with the public access terms of each source. No formal permission was required for noncommercial academic use.
