# Supplementary material for: Cardiovascular Disease as a Predictor of Dementia: A Cross‐National Ecological Study of 204 Countries
Source: Health Sci Rep. 2025 Aug 27;8(9):e71179. doi: 10.1002/hsr2.71179 (PMC12391024; doi:10.1002/hsr2.71179)
Supplement: Supplementary file 1 — SF 1 Table: Combined Correlation Matrix of Cardiovascular, Neurological, Demographic, and Socioeconomic Variables (Pearson correlations shown above the diagonal; Spearman correlations shown below the diagonal). [file HSR2-8-e71179-s001.docx]

SF 1 Table: Combined Correlation Matrix of Cardiovascular, Neurological, Demographic, and Socioeconomic Variables (Pearson correlations shown above the diagonal; Spearman correlations shown below the diagonal)

|  | Atrial fibrillation and flutter | Cardiomyopathy and myocarditis | Cardiovascular diseases | Endocarditis | Intracerebral hemorrhage | Ischemic heart disease | Ischemic stroke | Lower extremity peripheral arterial disease | Myocarditis | Non-rheumatic calcific aortic valve disease | Non-rheumatic degenerative mitral valve disease | Non-rheumatic valvular heart disease | Pulmonary Arterial Hypertension | Rheumatic heart disease | Stroke | Subarachnoid hemorrhage | Alzheimer's disease and other dementias | Ageing | Economic Affluence | Genetic Predisposition | Urban Advantage |
| --- | --- | --- | --- | --- | --- | --- | --- | --- | --- | --- | --- | --- | --- | --- | --- | --- | --- | --- | --- | --- | --- |
| Atrial fibrillation and flutter | 1 | 0.918^**^ | 0.689^**^ | 0.757^**^ | 0.023 | 0.322^**^ | 0.548^**^ | 0.889^**^ | 0.918^**^ | 0.778^**^ | 0.597^**^ | 0.754^**^ | 0.396^**^ | -0.659^**^ | 0.483^**^ | 0.472^**^ | 0.876^**^ | 0.713^**^ | 0.616^**^ | 0.608^**^ | 0.466^**^ |
| Cardiomyopathy and myocarditis | 0.955^**^ | 1 | 0.708^**^ | 0.783^**^ | 0.123 | 0.320^**^ | 0.643^**^ | 0.920^**^ | 1.000^**^ | 0.783^**^ | 0.660^**^ | 0.789^**^ | 0.359^**^ | -0.702^**^ | 0.600^**^ | 0.596^**^ | 0.927^**^ | 0.739^**^ | 0.631^**^ | 0.648^**^ | 0.466^**^ |
| Cardiovascular diseases | 0.801^**^ | 0.745^**^ | 1 | 0.525^**^ | 0.198^**^ | 0.868^**^ | 0.839^**^ | 0.685^**^ | 0.708^**^ | 0.631^**^ | 0.632^**^ | 0.685^**^ | 0.338^**^ | -0.681^**^ | 0.776^**^ | 0.440^**^ | 0.777^**^ | 0.616^**^ | 0.430^**^ | 0.622^**^ | 0.401^**^ |
| Endocarditis | 0.825^**^ | 0.811^**^ | 0.604^**^ | 1 | 0.013 | 0.194^**^ | 0.427^**^ | 0.811^**^ | 0.783^**^ | 0.628^**^ | 0.378^**^ | 0.557^**^ | 0.382^**^ | -0.591^**^ | 0.382^**^ | 0.482^**^ | 0.781^**^ | 0.669^**^ | 0.610^**^ | 0.547^**^ | .448^**^ |
| Intracerebral hemorrhage | 0.124 | 0.214^**^ | 0.155^*^ | 0.128 | 1 | 0.104 | 0.308^**^ | 0.073 | 0.123 | -0.107 | 0.131 | 0.003 | 0.156^*^ | -0.074 | 0.600^**^ | 0.423^**^ | 0.028 | -0.045 | -0.240^**^ | 0.118 | -0.144^*^ |
| Ischemic heart disease | 0.521^**^ | 0.423^**^ | 0.879^**^ | 0.312^**^ | 0.075 | 1 | 0.639^**^ | 0.285^**^ | 0.320^**^ | 0.283^**^ | 0.344^**^ | 0.338^**^ | 0.158^*^ | -0.530^**^ | 0.569^**^ | 0.221^**^ | 0.422^**^ | 0.420^**^ | 0.222^**^ | 0.490^**^ | 0.286^**^ |
| Ischemic stroke | 0.772^**^ | 0.780^**^ | 0.876^**^ | 0.653^**^ | 0.372^**^ | 0.662^**^ | 1 | 0.581^**^ | 0.643^**^ | 0.528^**^ | 0.576^**^ | 0.597^**^ | 0.378^**^ | -.553^**^ | 0.945^**^ | 0.461^**^ | 0.685^**^ | 0.422^**^ | 0.263^**^ | 0.455^**^ | 0.246^**^ |
| Lower extremity peripheral arterial disease | 0.943^**^ | 0.933^**^ | 0.834^**^ | 0.838^**^ | 0.152^*^ | 0.533^**^ | 0.822^**^ | 1 | 0.920^**^ | 0.808^**^ | 0.636^**^ | 0.791^**^ | 0.333^**^ | -0.726^**^ | 0.528^**^ | 0.494^**^ | 0.928^**^ | 0.752^**^ | 0.673^**^ | 0.625^**^ | 0.520^**^ |
| Myocarditis | 0.955^**^ | 1.000^**^ | 0.745^**^ | 0.811^**^ | 0.214^**^ | 0.423^**^ | 0.780^**^ | 0.933^**^ | 1 | 0.783^**^ | 0.660^**^ | 0.789^**^ | 0.359^**^ | -0.702^**^ | 0.600^**^ | 0.596^**^ | 0.927^**^ | 0.739^**^ | 0.631^**^ | 0.648^**^ | 0.466^**^ |
| Non-rheumatic calcific aortic valve disease | 0.900^**^ | 0.868^**^ | 0.829^**^ | 0.734^**^ | -0.053 | 0.599^**^ | 0.728^**^ | 0.896^**^ | 0.868^**^ | 1 | 0.695^**^ | 0.933^**^ | 0.278^**^ | -0.579^**^ | 0.416^**^ | 0.325^**^ | 0.833^**^ | 0.631^**^ | 0.647^**^ | 0.470^**^ | 0.420^**^ |
| Non-rheumatic degenerative mitral valve disease | 0.820^**^ | 0.857^**^ | 0.840^**^ | 0.650^**^ | 0.216^**^ | 0.601^**^ | 0.832^**^ | 0.890^**^ | 0.857^**^ | 0.865^**^ | 1 | 0.907^**^ | 0.196^**^ | -0.478^**^ | 0.535^**^ | 0.370^**^ | 0.674^**^ | 0.481^**^ | 0.457^**^ | 0.404^**^ | 0.281^**^ |
| Non-rheumatic valvular heart disease | 0.900^**^ | 0.898^**^ | 0.853^**^ | 0.726^**^ | 0.065 | 0.605^**^ | 0.795^**^ | 0.920^**^ | 0.898^**^ | 0.974^**^ | 0.947^**^ | 1 | 0.261^**^ | -0.578^**^ | 0.511^**^ | 0.375^**^ | 0.825^**^ | 0.609^**^ | 0.607^**^ | 0.476^**^ | 0.387^**^ |
| Pulmonary Arterial Hypertension | 0.367^**^ | 0.364^**^ | 0.327^**^ | 0.318^**^ | 0.237^**^ | 0.165^*^ | 0.419^**^ | 0.334^**^ | 0.364^**^ | 0.207^**^ | 0.211^**^ | 0.219^**^ | 1 | -0.105 | 0.369^**^ | 0.162^*^ | 0.371^**^ | 0.123 | 0.095 | 0.102 | 0.032 |
| Rheumatic heart disease | -0.679^**^ | -0.663^**^ | -0.705^**^ | -0.616^**^ | 0.071 | -0.587^**^ | -0.625^**^ | -0.777^**^ | -0.663^**^ | -0.752^**^ | -0.754^**^ | -0.761^**^ | -0.098 | 1 | -0.505^**^ | -0.494^**^ | -.700^**^ | -0.809^**^ | -0.678^**^ | -0.768^**^ | -0.590^**^ |
| Stroke | 0.696^**^ | 0.738^**^ | 0.773^**^ | 0.593^**^ | 0.642^**^ | 0.561^**^ | 0.929^**^ | 0.744^**^ | 0.738^**^ | 0.605^**^ | 0.782^**^ | 0.698^**^ | 0.414^**^ | -0.500^**^ | 1 | 0.573^**^ | 0.598^**^ | 0.362^**^ | 0.157^*^ | 0.443^**^ | 0.172^*^ |
| Subarachnoid hemorrhage | 0.668^**^ | 0.675^**^ | 0.520^**^ | 0.611^**^ | 0.423^**^ | 0.354^**^ | 0.564^**^ | 0.665^**^ | 0.675^**^ | 0.616^**^ | 0.611^**^ | 0.639^**^ | 0.199^**^ | -0.479^**^ | 0.661^**^ | 1 | 0.492^**^ | 0.500^**^ | 0.248^**^ | 0.579^**^ | 0.272^**^ |
| Alzheimer's disease and other dementias | 0.945^**^ | 0.916^**^ | 0.868^**^ | 0.802^**^ | 0.066 | 0.597^**^ | 0.823^**^ | 0.959^**^ | 0.916^**^ | 0.924^**^ | 0.863^**^ | 0.928^**^ | 0.355^**^ | -0.743^**^ | 0.709^**^ | 0.620^**^ | 1 | 0.743^**^ | 0.605^**^ | 0.609^**^ | 0.505^**^ |
| Ageing | 0.787^**^ | 0.751^**^ | 0.719^**^ | 0.723^**^ | -0.204^**^ | 0.534^**^ | 0.594^**^ | 0.826^**^ | 0.751^**^ | 0.858^**^ | 0.757^**^ | 0.836^**^ | 0.097 | -0.824^**^ | 0.446^**^ | 0.516^**^ | 0.832^**^ | 1 | 0.733^**^ | 0.876^**^ | 0.604^**^ |
| Economic Affluence | 0.757^**^ | 0.734^**^ | 0.707^**^ | 0.689^**^ | -0.228^**^ | 0.542^**^ | 0.605^**^ | 0.793^**^ | 0.734^**^ | 0.851^**^ | 0.742^**^ | 0.825^**^ | 0.145^*^ | -0.812^**^ | 0.434^**^ | 0.449^**^ | 0.779^**^ | 0.880^**^ | 1 | 0.567^**^ | 0.649^**^ |
| Genetic Predisposition | 0.830^**^ | 0.811^**^ | 0.753^**^ | 0.761^**^ | -0.104 | 0.542^**^ | 0.653^**^ | 0.871^**^ | 0.811^**^ | 0.900^**^ | 0.814^**^ | 0.883^**^ | 0.154^*^ | -0.825^**^ | 0.527^**^ | 0.561^**^ | 0.853^**^ | 0.930^**^ | 0.895^**^ | 1 | 0.523^**^ |
| Urban Advantage | 0.486^**^ | 0.452^**^ | 0.460^**^ | 0.495^**^ | -0.263^**^ | 0.355^**^ | 0.365^**^ | 0.539^**^ | 0.452^**^ | 0.587^**^ | 0.454^**^ | 0.540^**^ | 0.002 | -0.612^**^ | 0.223^**^ | 0.296^**^ | 0.530^**^ | 0.640^**^ | 0.720^**^ | 0.630^**^ | 1.000 |

Sample sizes for correlation analyses ranged from 185 to 204.
*Correlation is significant at the 0.05 level (2-tailed); **Correlation is significant at the 0.01 level (2-tailed).

Aging in females was measured by life expectancy at birth (World Bank, 2018).
Economic affluence was represented by GDP per capita in purchasing power parity (PPP), also from the World Bank (2018).
Genetic predisposition was indexed using the Biological State Index (Ibs), reflecting the accumulation of dementia-related genetic risk due to relaxed natural selection (You & Henneberg, 2022).
Urban living was measured by the percentage of the population residing in urban areas (World Bank, 2018).
